# AGE DIFFERENCES IN DEPRESSION AND ANXIETY LEVELS OF OPEN HEART SURGERY PATIENTS

**DOI:** 10.1093/geroni/igac059.3050

**Published:** 2022-12-20

**Authors:** Crim Sabuncu, Amy Ai

**Affiliations:** Florida State University, Tallahassee, Florida, United States; Florida State University, Tallahassee, Florida, United States

## Abstract

Age, as well as gender, are key demographic measures in cardiac patients. Further, depression and anxiety have been long established to be comorbidities of heart disease (HD). In this prospective study, to examine the impact of age and gender in preoperative depression and postoperative anxiety symptoms of HD patients receiving open heart surgery (OHS), we collected two waves of survey data along with medical indices from patients at a top-10 heart center in the USA (n = 481, mean age = 62.18 ± 12.04, female 42%). The collected survey data included socio-demographic information (age, gender, race), religious affiliation and other religious factors, general health and health behaviors, medical comorbidities, cardiovascular health indices, dispositional optimism, hope, social support, depression symptoms, and anxiety symptoms. Statistical analysis was carried out using hierarchical linear regression analyses. Findings support that higher age correlates with increased levels of cardiac symptoms. Further, younger age was associated with higher levels of postoperative depression, while older age was associated with higher levels of postoperative anxiety. Higher levels of dispositional optimism were consistently associated with lower levels of both postoperative depression and postoperative anxiety regardless of age. Female gender had an impact on depression scores but not on anxiety scores. Findings suggest that medical practitioners might benefit from being more attentive to non-medical conditions such as mood states, dispositional optimism, and general positive expectations about the future in post-OHS life. The findings also suggest that there might be age and gender differences in expected postoperative psychosocial symptomology for OHS patients.

